# Circulating mitochondrial DNA is an early indicator of severe illness and mortality from COVID-19

**DOI:** 10.1172/jci.insight.143299

**Published:** 2021-02-22

**Authors:** Davide Scozzi, Marlene Cano, Lina Ma, Dequan Zhou, Ji Hong Zhu, Jane A. O’Halloran, Charles Goss, Adriana M. Rauseo, Zhiyi Liu, Sanjaya K. Sahu, Valentina Peritore, Monica Rocco, Alberto Ricci, Rachele Amodeo, Laura Aimati, Mohsen Ibrahim, Ramsey Hachem, Daniel Kreisel, Philip A. Mudd, Hrishikesh S. Kulkarni, Andrew E. Gelman

**Affiliations:** 1 Division of Cardiothoracic Surgery, Department of Surgery;; 2 Division of Pulmonary and Critical Care Medicine, Department of Medicine;; 3 Division of Infectious Diseases, Department of Medicine; and; 4 Division of Biostatistics, Washington University School of Medicine, St. Louis, Missouri, USA.; 5 Division of Thoracic Surgery and; 6 Division of Anesthesiology, Department of Medical-Surgical Science and Translational Medicine, Sapienza University of Rome, Rome, Italy.; 7 Division of Pulmonology, Department of Clinical and Molecular Medicine, Sapienza University of Rome, Rome, Italy.; 8 Laboratory Analysis-Flow Cytometry Section, Sapienza University of Rome, Rome, Italy.; 9 Division of Emergency Medicine,; 10 Department of Molecular Microbiology, and; 11 Department of Pathology & Immunology, Washington University School of Medicine, St. Louis, Missouri, USA.

**Keywords:** COVID-19, Immunology, Complement, Mitochondria

## Abstract

**Background:**

Mitochondrial DNA (MT-DNA) are intrinsically inflammatory nucleic acids released by damaged solid organs. Whether circulating cell-free MT-DNA quantitation could be used to predict the risk of poor COVID-19 outcomes remains undetermined.

**Methods:**

We measured circulating MT-DNA levels in prospectively collected, cell-free plasma samples from 97 subjects with COVID-19 at hospital presentation. Our primary outcome was mortality. Intensive care unit (ICU) admission, intubation, vasopressor, and renal replacement therapy requirements were secondary outcomes. Multivariate regression analysis determined whether MT-DNA levels were independent of other reported COVID-19 risk factors. Receiver operating characteristic and area under the curve assessments were used to compare MT-DNA levels with established and emerging inflammatory markers of COVID-19.

**Results:**

Circulating MT-DNA levels were highly elevated in patients who eventually died or required ICU admission, intubation, vasopressor use, or renal replacement therapy. Multivariate regression revealed that high circulating MT-DNA was an independent risk factor for these outcomes after adjusting for age, sex, and comorbidities. We also found that circulating MT-DNA levels had a similar or superior area under the curve when compared against clinically established measures of inflammation and emerging markers currently of interest as investigational targets for COVID-19 therapy.

**Conclusion:**

These results show that high circulating MT-DNA levels are a potential early indicator for poor COVID-19 outcomes.

**Funding:**

Washington University Institute of Clinical Translational Sciences COVID-19 Research Program and Washington University Institute of Clinical Translational Sciences (ICTS) NIH grant UL1TR002345.

## Introduction

Coronavirus disease 2019 (COVID-19) is a respiratory tract infection caused by severe acute respiratory syndrome coronavirus 2 (SARS-CoV-2) that has resulted in a global health emergency, causing considerable strain on economic, social, and medical systems (1). COVID-19 presents in a wide spectrum of severity. Although most patients develop only mild or uncomplicated illness, others require prolonged hospitalization, ICU care, and intubation for respiratory support. In severe cases, patients can develop acute respiratory distress syndrome (ARDS), cytokine storm, multiorgan failure, and death (1). Although the underlying mechanisms of severe COVID-19 illness remain unclear, it appears to be exacerbated by an overexuberant innate immune response (2). These observations have led to several ongoing clinical trials targeting components of the innate immune response, such as inflammatory cytokine signaling and complement activation (3–5).

Previous work has established that viral infection can trigger cellular necrosis, which in turn inhibits viral replication along with amplifying antiviral immune responses through the release of damage-associated molecular patterns (DAMPs) (6). DAMPs in particular are potent triggers of innate responses through their engagement of pattern recognition receptors, such as Toll-like receptors (TLRs), that drive the expression of inflammatory cytokines and presentation of viral antigens (7). Mitochondrial DNA (MT-DNA) is a member of a group of mitochondrial DAMPs (MT-DAMPs) released by injured or dying cells and is recognized by TLR9 because of encoded hypomethylated CpG motifs reminiscent of an ancestral bacterial origin (8). MT-DNA levels have been previously shown to be elevated in the plasma of patients who develop ARDS and multiorgan dysfunction during sepsis, as well as during sterile injury, including trauma, hemorrhagic shock, and ischemia/reperfusion (9–14). The release of MT-DNA is often accompanied by the release of other MT-DAMPs, such as N-formylated peptides, cytochrome *c*, and cardiolipin, which collectively engage multiple TLRs and the N-formylated peptide receptor-1 that in turn induce not only inflammatory cytokine expression (15–17) but also the generation of reactive oxygen species and the facilitation of neutrophil trafficking and activation (14, 18, 19). Through these effects, MT-DAMPs can directly contribute to acute lung injury and systemic inflammation (8). Given that ARDS secondary to SARS-CoV-2 infection is also linked with lung tissue injury and immune cell activation (20), we asked if elevated levels of circulating MT-DNA could be used as a risk indicator for the development of severe illness.

Here, we demonstrate that COVID-19 patients with high circulating levels of cell-free MT-DNA at the time of hospital presentation are more likely to require intensive care unit (ICU) care and intubation and are at heightened risk of death. In addition, MT-DNA quantitation as predictor of poor COVID-19 outcomes is comparable to or better than inflammation indicators commonly used in current clinical practice, as well as certain emerging immune markers.

## Results

### Participants.

**Figure 1:**
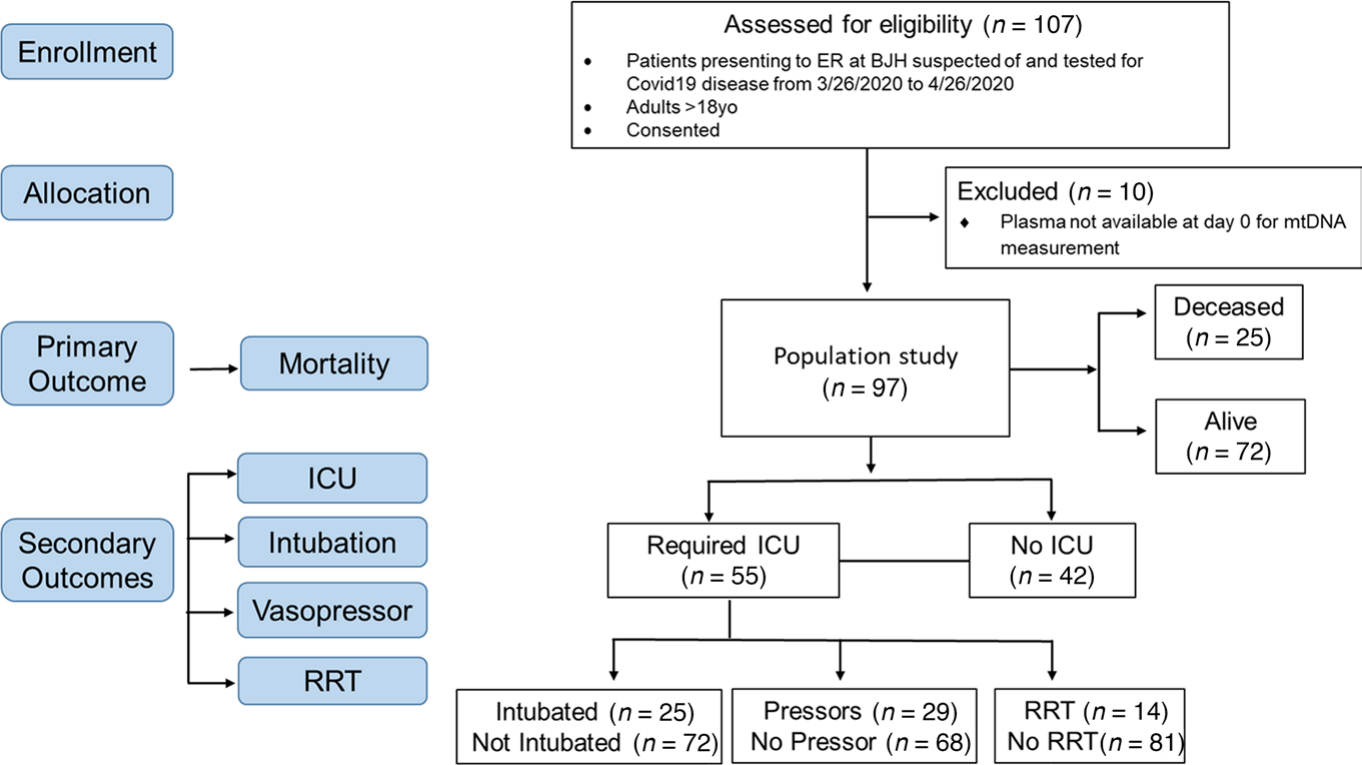
CONSORT flow diagram showing enrollment of patients, allocation, and outcomes. RRT, renal replacement therapy.

A total of 107 subjects were assessed for eligibility from March 26, 2020, to April 26, 2020. Of these, 97 adult subjects with laboratory-confirmed COVID-19 were included in our study (Table 1 and Figure 1). Ten subjects were excluded because they did not have samples from the day of presentation. The median age of the population in our study was 65 (interquartile range 54–73). Among these subjects, 55.7% (54/97) were male and 44.3% (43/97) were female; 77.3% (75/97) were African American, 20.6% (20/97) were White, 1% (1/97) was Middle Eastern, and 1% (1/97) was Indian; and 46.4% (45/97) were obese (BMI ≥ 30), while 49.5% (48/97) were nonobese (BMI 18.5–29.9) and 4.1% (4/97) were underweight (BMI < 18.5). A positive smoking history was reported in 46.4% (45/97) of subjects. The median follow-up time was 81 days (interquartile range 74–87).

### Comorbidities and outcomes.

The primary outcome of mortality was observed in 25.8% (25/97) of subjects in our study (Table 1). Those who died were older [76.2 versus 61.1 years, OR 1.08 (1.04–1.13), *P* = 0.0003], were more likely to be smokers [64% versus 40.3%, OR 2.63 (1.04–7.00), *P* = 0.04], and were more likely to have type 2 diabetes mellitus [72% versus 45.8%, OR 3.04 (1.17–8.64), *P* = 0.02], coronary artery disease [48% versus 19.4%, OR 3.8 (1.44–10.34), *P* = 0.007], and 2 or more comorbidities [84% versus 56.9%, OR 5.54 (1.72–24.91, *P* = 0.009] on univariate analysis (Table 1). The time to discharge for subjects who survived was mean 12.4 days, median 7 days (interquartile range 3–18), and range 1–56.

Of the subjects with COVID-19, 56.7% (55/97, Table 2) required an ICU admission. These subjects were older [71 versus 54.4, OR 1.1 (1.06–1.15), *P* < 0.0001] and were more likely to have type 2 diabetes mellitus [65.5% versus 35.7%, OR 3.41 (1.49–8.07), *P* = 0.004] and 2 or more comorbidities [72.7% versus 52.4%, OR 2.66 (1.14–6.38), *P* = 0.02]. As expected, the time to discharge was higher for patients requiring ICU admission [15 days (interquartile range 5–28), *n* = 27; versus 5 days (interquartile range 3–12.5), *n* = 36, *P* = 0.0012]. Among patients, 25.8% (25/97) required invasive mechanical ventilation as a treatment for acute respiratory failure. The main clinical characteristic associated with higher risk for intubation was age, with a median of 70 years for intubated subjects compared with 61 years for nonintubated subjects [OR 1.05 (1.01–1.09), *P* = 0.005] on univariate analysis (Table 3). As expected, the time to discharge was higher for patients who required intubation [27.5 days (interquartile range 18.5–38.5), *n* = 12; versus 5 days (interquartile range 3–13), *n* = 51, *P* < 0.0001].

### COVID-19 patients with high circulating MT-DNA are at higher risk for mortality.

**Figure 2:**
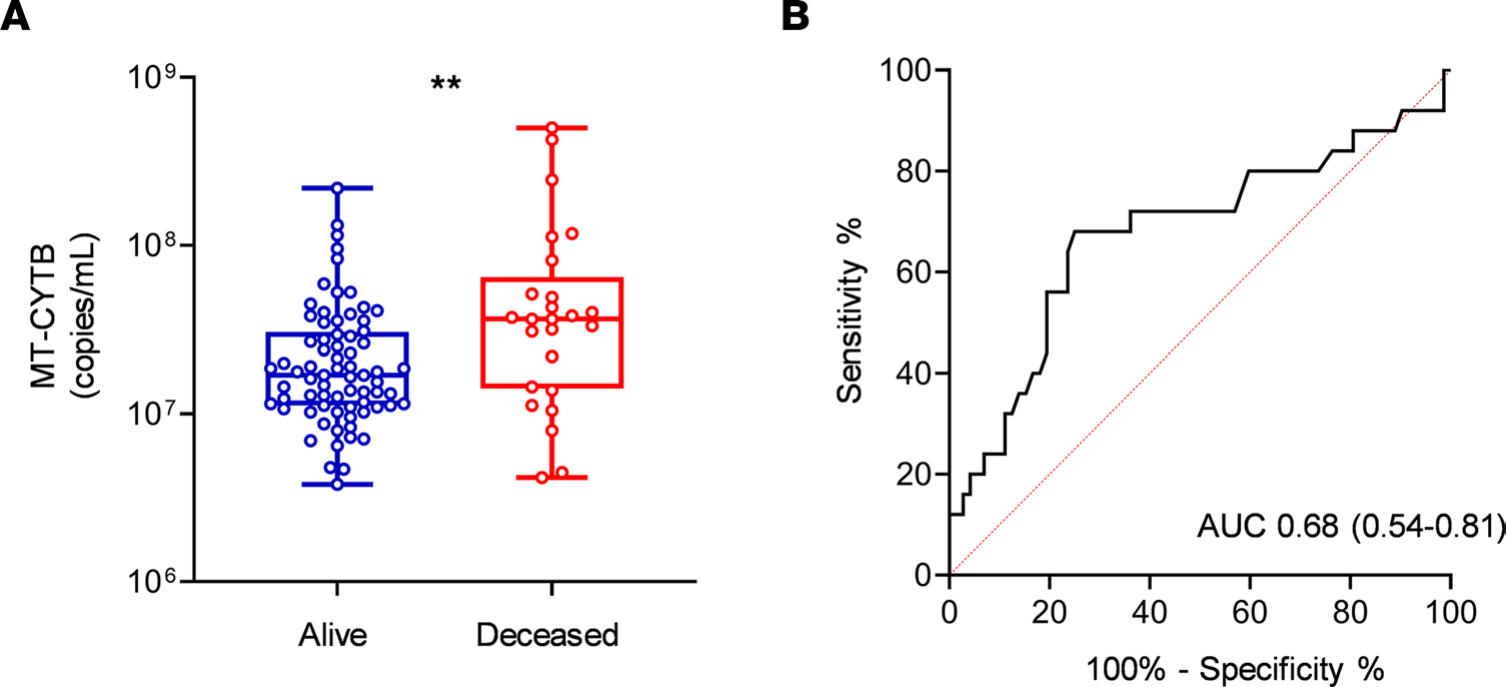
High circulating MT-DNA levels predict a higher risk of mortality in COVID-19 patients. Plasma for determination of circulating levels of MT-CYTB was obtained at time of hospital presentation. (**A**) Box-and-whisker plots of MT-CYB levels in relation to mortality status in COVID-19 patients. The box plots depict the minimum and maximum values (whiskers), the upper and lower quartiles, and the median. The length of the box represents the interquartile range. (**B**) Receiver operating characteristic (ROC) curves in predicting the outcome mortality based on MT-CYTB levels. Statistical significance was determined using Mann-Whitney *U* test (**P < 0.01).

To assess MT-DNA levels in COVID-19 patients, we utilized a previously established in situ quantitative PCR method (14) to measure the accumulation of fragments derived from the mitochondrial encoded gene cytochrome *b* (MT-CYTB) within cell-free circulating plasma. Plasma MT-CYTB levels were elevated in those subjects who died from COVID-19 [7.56 (7.15–7.81, *n* = 25)] compared with those who survived [7.23 (7.050–7.485), *n* = 72, *P* = 0.008, Figure 2A] and were associated with an increased risk for mortality on univariate logistic regression (OR 2.24, 95% CI 1.29–4.16, *P* = 0.006). For MT-CYTB, area under the curve (AUC) for mortality was 0.68 (95% CI 0.54–0.81, Figure 2B). On multivariable logistic regression, plasma MT-CYTB levels remained an independent risk factor for mortality when adjusted for age, sex, and 2 or more comorbidities (OR_adj_, 2.19, 95% CI 1.19–4.28, *P* = 0.015, Table 4). Levels of mitochondrial cytochrome *c* oxidase III (MT-COX3), another MT-DNA–encoded gene (Supplemental Figure 1B; supplemental material available online with this article; https://doi.org/10.1172/jci.insight.143299DS1), revealed an increased trend in subjects who died [5.63 (5.00–6.46), *n* = 25] relative to those who survived [5.32 (4.90–5.83), *n* = 72] but did not reach statistical significance (*P* = 0.1).

### COVID-19 patients with high circulating MT-DNA are more likely to require ICU admission and intubation.

**Figure 3:**
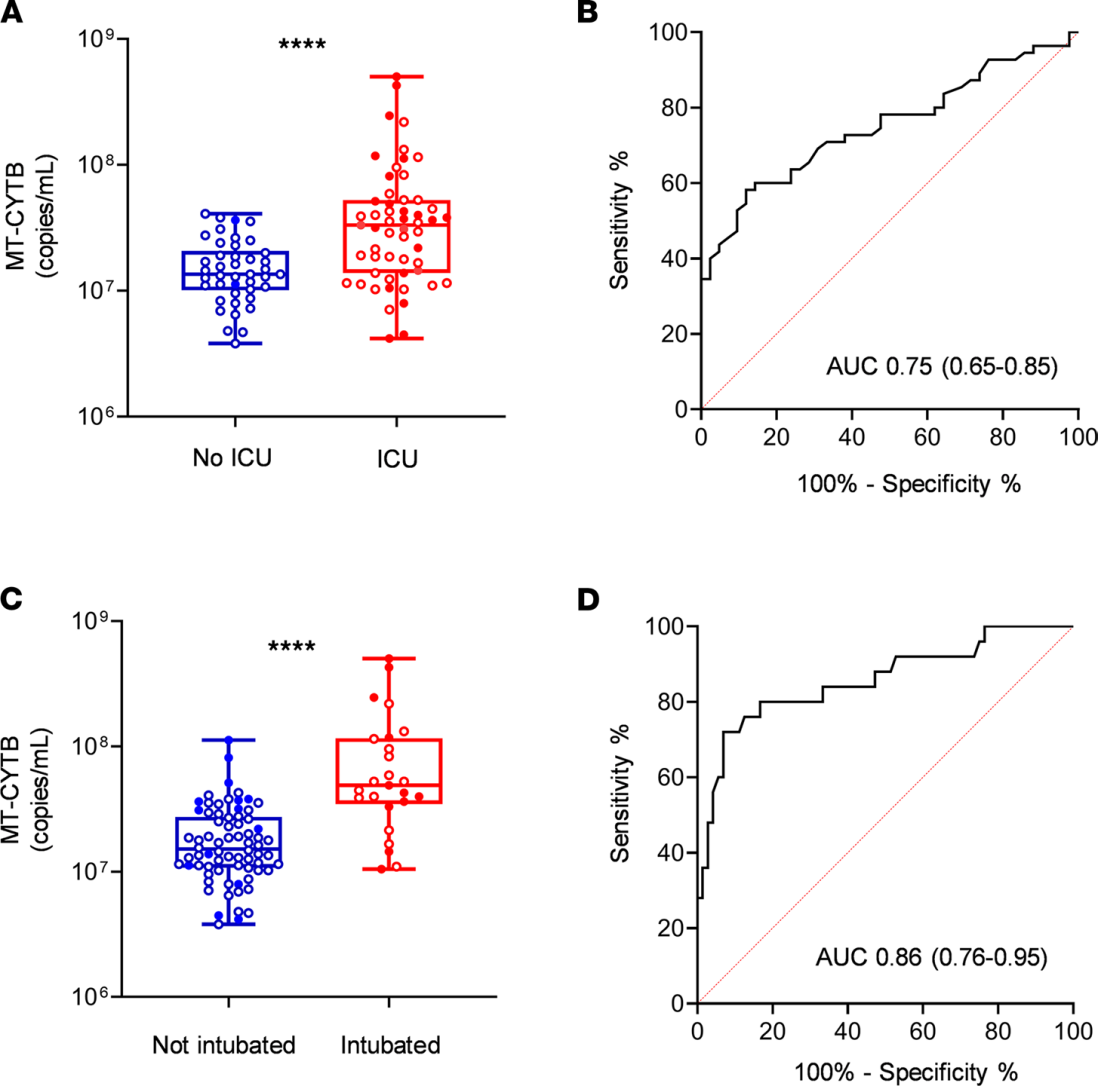
High circulating MT-DNA levels predict a higher risk of ICU requirement and intubation in COVID-19 patients. Plasma for determination of circulating levels of MT-CYTB was obtained at time of hospital presentation. Box-and-whisker plots of MT-CYB levels in relation to (**A**) ICU admission and (**C**) intubation in COVID-19 patients. The box plots depict the minimum and maximum values (whiskers), the upper and lower quartiles, and the median. The length of the box represents the interquartile range. Empty dots indicate alive patients and shaded dots indicate deceased patients. ROC curves in predicting the outcome (**B**) ICU and (**D**) intubation based on MT-CYTB levels. Statistical significance was determined using Mann-Whitney *U* test (****P <.0001).

Plasma MT-CYTB levels were elevated in those subjects with COVID-19 who required an ICU admission [7.52 (7.14–7.72), *n* = 55] compared with those who were not admitted to the ICU [7.13 (7.00–7.31), *P* < 0.0001, *n* = 42, Figure 3A]. MT-CYTB levels were associated with ICU admission on univariate logistic regression (OR 4.25, 95% CI 2.15–9.59, *P* = 0.0001). For MT-CYTB, AUC for ICU admission was 0.75 (95% CI 0.65–0.85, Figure 3B). On multivariable logistic regression, plasma MT-CYTB levels remained an independent risk factor for ICU admission after adjusting for age, sex, and 2 or more comorbidities (OR_adj_, 3.97, 95% CI 1.83–10.34, *P* = 0.002, Table 5). Notably, we also made similar findings for MT-COX3 [Supplemental Figure 1C; OR_adj_ for age, sex, and 2 or more comorbidities, 1.47; 95% CI 1.14–1.95; *P* = 0.005; AUC of COX3 for ICU admission 0.69 (95% CI 0.58–0.79)].

Additionally, plasma MT-CYTB levels were elevated in those subjects with COVID-19 who required intubation [7.69 (7.54–8.07, *n* = 25)] versus those who did not require intubation [7.18 (7.033–7.438), *n* = 72, *P* < 0.0001, Figure 3C]. Plasma MT-CYTB levels were associated with intubation on univariate logistic regression (OR 9.12, 95% 3.77–28.56, *P* < 0.0001). For MT-CYTB, AUC for intubation was 0.86 (95% CI 0.76–0.95, Figure 3D). On multivariable logistic regression, plasma MT-CYTB levels remained an independent risk factor for intubation (OR_adj_, 8.47, 95% CI 3.49–27.33, *P* < 0.0001, Table 6). Similar findings were observed with MT-COX3 [Supplemental Figure 1D; OR_adj_ for age, sex, and 2 or more comorbidities, 2.69; 95% CI 1.77–4.74; *P* < 0.0001; AUC for intubation 0.80 (95% CI 0.69–0.91)].

### COVID-19 patients with high circulating MT-DNA are more likely to require vasopressors and renal replacement therapy.

**Figure 4:**
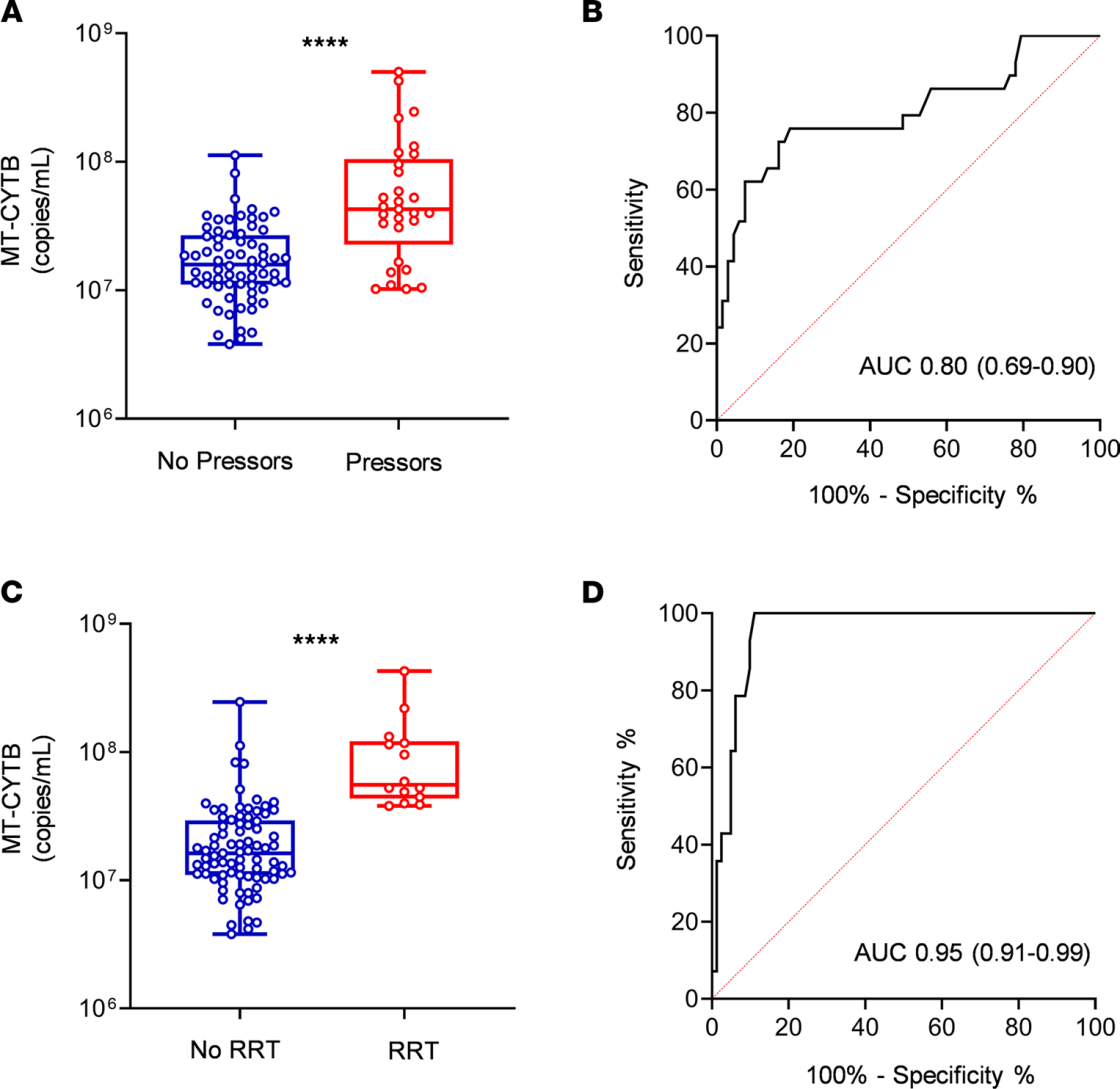
High circulating MT-DNA levels predict a higher risk for end organ dysfunction requiring vasopressors and renal replacement in COVID-19 patients. Plasma for determination of circulating levels of MT-CYTB was obtained at time of hospital presentation. Box-and-whisker plots of MT-CYB levels in relation to (**A**) requirement for vasopressors and (**C**) renal replacement in COVID-19 patients. The box plots depict the minimum and maximum values (whiskers), the upper and lower quartiles, and the median. The length of the box represents the interquartile range. ROC curves in predicting the outcomes (**B**) requirement for pressors and (**D**) renal replacement based on MT-CYTB levels. Statistical significance was determined using Mann-Whitney *U* test (****P < 0.0001).

Plasma MT-CYTB levels were also elevated in those subjects with COVID-19 who ultimately had end organ dysfunction requiring vasopressors [7.63 (7.35–8.02), *n* = 29] compared with those who did not require vasopressors [7.20 (7.04–7.43), *P* < 0.0001, *n* = 68, Figure 4A]. For MT-CYTB, AUC for vasopressor requirement was 0.80 (95% CI 0.69–0.91, Figure 4B). Similarly, plasma MT-COX3 levels were elevated in subjects requiring vasopressors [5.98 (5.25–6.68), *n* = 29; compared with 5.25 (4.85–5.70), *P* = 0.0001, *n* = 68 (Supplemental Figure 1E); AUC of COX3 for vasopressor requirement was 0.74 (95% CI 0.62–0.86)]. Notably, plasma MT-CYTB levels on admission were also elevated in subjects who had end organ dysfunction requiring RRT while in the ICU [7.75 (7.64–8.08), *n* = 14] compared with those who did not require RRT [7.2 (7.04–7.47), *P* < 0.0001, *n* = 81, Figure 4C]. For MT-CYTB, AUC for RRT requirement was 0.95 (95% CI 0.91–0.99, Figure 4D). Similarly, plasma MT-COX3 levels were elevated in subjects who required RRT [6.59 (6.36–7.12), *n* = 14] compared with those who did not require RRT [5.24 (4.81–5.69), *P* < 0.0001, *n* = 81] (Supplemental Figure 1F); AUC of COX3 for RRT requirement was 0.94 (95% CI 0.88–0.99)].

### Circulating MT-DNA levels show similar or improved sensitivity over clinically established measurements of inflammation used in COVID-19 patients.

**Figure 5:**
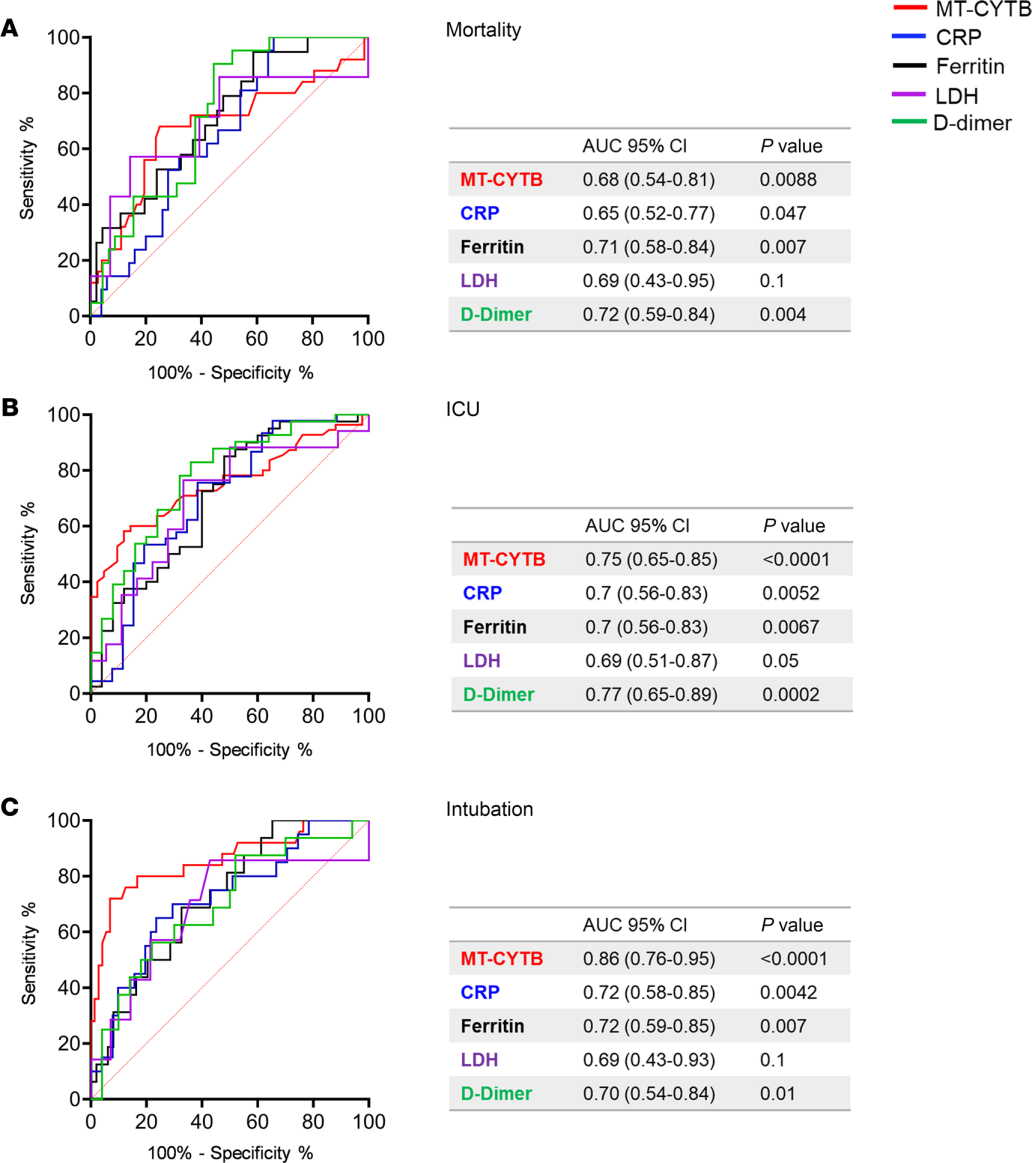
Circulating MT-DNA levels have similar or improved accuracy over clinically utilized biomarkers for outcomes of severity in COVID-19. Blood samples for determination of biomarkers levels were collected within 24 hours from hospital presentation. ROC curves in predicting the outcomes (**A**) mortality, (**B**) admission to ICU, and (**C**) intubation based on MT-CYTB (red), reactive C protein (CRP) (blue), ferritin (black), lactic acid dehydrogenase (LDH) (purple), and D-dimer (green) levels. AUC with 95% CI and *P* values for the different biomarkers are summarized in the corresponding tables.

Plasma MT-CYTB levels had a similar AUC for mortality when compared with lactic acid dehydrogenase (LDH), ferritin, or D-dimer levels and were better than C-reactive protein (CRP) drawn within the first 24 hours of presentation (Figure 5A). Importantly, the AUC for plasma MT-CYTB levels was superior to CRP, LDH, ferritin, and D-dimer levels when predicting the need for an ICU admission (Figure 5B) or intubation (Figure 5C). A similar pattern was identified for MT-COX3 when compared with CRP, LDH, ferritin, and D-dimer levels for predicting the need for an ICU admission (Supplemental Figure 2B) and inferior for predicting mortality (Supplemental Figure 2A) but was superior to these clinically utilized markers for the need for intubation (Supplemental Figure 2C).

### Circulating MT-DNA levels correlate with other emerging markers of COVID-19 severity.

**Figure 6:**
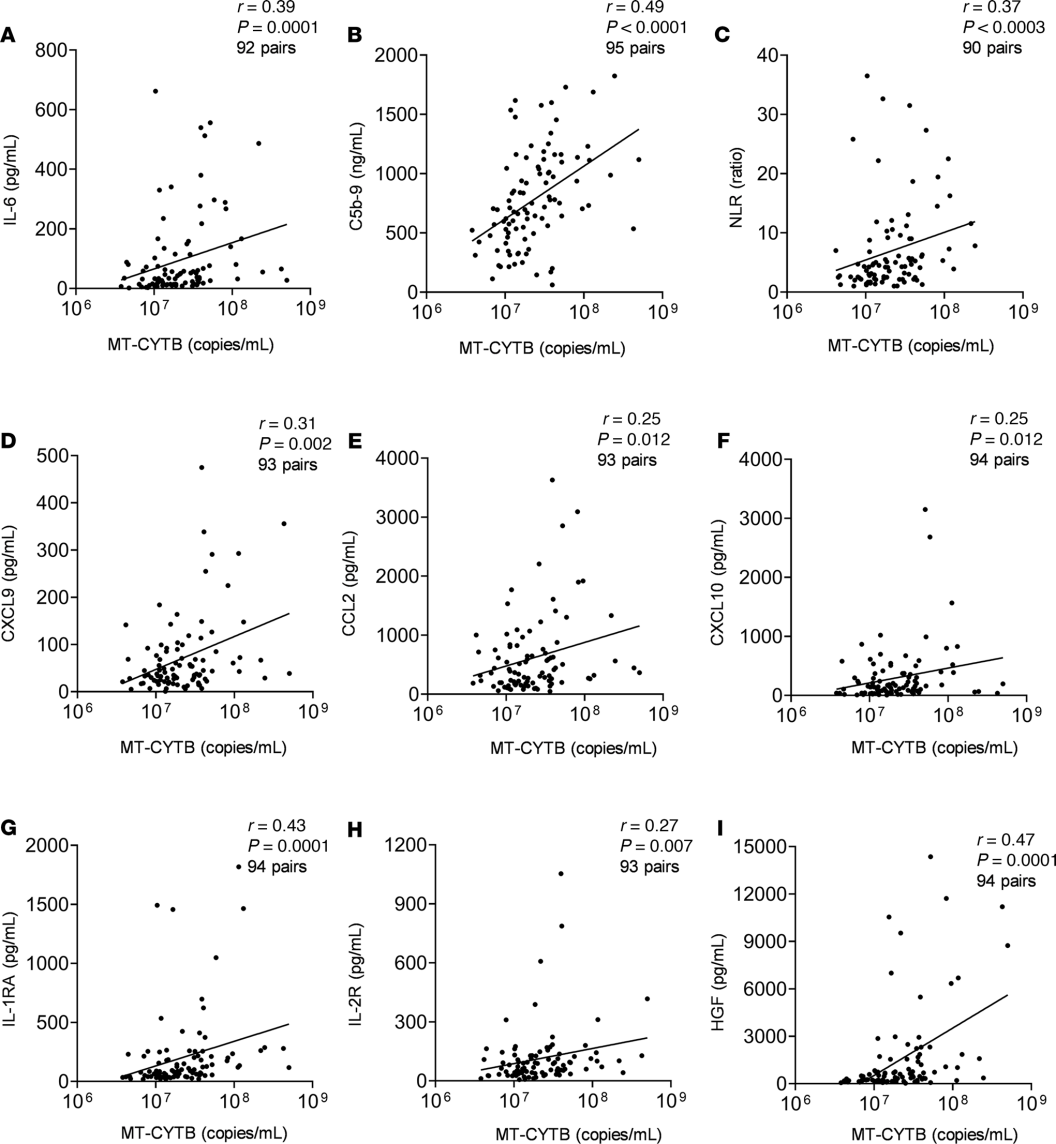
Circulating MT-DNA levels correlate with other emerging markers of inflammation and cytokines in COVID-19 patients. Scatter plots showing the correlation between MT-CYTB and (**A**) IL-6, (**B**) C5b-9 (terminal complement complex), and (**C**) neutrophil-to-lymphocyte ratio (NLR), (**D**) CXCL9, (**E**) CCL2, (**F**) CXCL10, (**G**) IL-1RA, (**H**) IL-2R, and (**I**) HGF. The degree of correlation was assessed using Spearman’s rank correlation coefficient test.

Plasma MT-CYTB levels moderately correlated with concurrently measured levels of IL-6, which has been implicated in the pathogenesis of COVID-19 (*r* = 0.39, 95% CI 0.196–0.555, *P* = 0.0001, *n* = 92, Figure 6A). Similarly, MT-CYTB levels highly correlated with plasma soluble C5b-9, which is a marker of complement activation and suggests the formation of a membrane attack complex (MAC) (*r* = 0.49, 95% CI 0.32–0.64, *P* < 0.0001, *n* = 95, Figure 6B). MT-CYTB also correlated with the neutrophil-to-lymphocyte ratio (*r* = 0.37, 95% CI 0.17–0.54, *P* = 0.0003, *n* = 90, Figure 6C). Plasma MT-CYTB levels had a similar or improved accuracy compared with IL-6 for mortality (Supplemental Figure 3A), ICU admission (Supplemental Figure 3B), and intubation (Supplemental Figure 3C).

We also observed significant correlations with other biomarkers that have been implicated in the pathogenesis of COVID-19, such as CXCL9 (*r* = 0.31, 95% CI 0.11–0.49, *P* = 0.002, *n* = 93, Figure 6D), CCL2 (*r* = 0.25, 95% CI 0.05–0.44, *P* = 0.01, *n* = 93, Figure 6E), CXCL10 (*r* = 0.25, 95% CI 0.05–0.44, *P* = 0.01, *P* = 94, Figure 6F), IL-1RA (*r* = 0.43, 95% CI 0.25–0.59, *P* < 0.0001, *n* = 94, Figure 6G), and IL-2R (*r* = 0.27, 95% CI 0.07– 0.46, *P* = 0.007, *P* = 93, Figure 6H). Similarly, the levels of HGF highly correlated with MT-CYTB in COVID-19 (*r* = 0.47, 95% CI 0.29–0.62, *P* < 0.0001, *n* = 94, Figure 6I).

## Discussion

Here we observe that high levels of circulating MT-DNA are an early independent risk factor for severe illness and mortality in hospitalized patients with COVID-19, after adjusting for age, sex, and comorbidities. Importantly, we made similar findings for 2 MT-DNA genes, MT-CYTB and MT-COX3, although the latter target did not reach significance for mortality risk. The reasons for this incongruence are not clear. One possibility is the accumulation of heteroplasmic mitochondrial genome deletions or point mutations that could prevent or attenuate PCR primer recognition (21). Heteroplasmic DNA deletions have been repeatedly demonstrated to accumulate with age within the MT-COX3 gene (22–24), and to a lesser degree, within the MT-CYTB locus, due to age-related defects of mitochondrial DNA polymerase proofreading capability (25, 26).

Although our studies were not specifically designed to identify the mechanisms that drive peripheral blood MT-DNA accumulation, significant correlations between LDH and IL-6 with MT-DNA levels point to a potential deleterious role for cellular necrosis in COVID-19 pathophysiology. LDH release and IL-6 production are reported indicators of cellular necrosis (27, 28). A specific form of necrosis, necroptosis, has been demonstrated to induce the release of damaged mitochondria (29). Necroptosis is characterized by the eventual loss of plasma membrane integrity due to the kinase activity of the receptor-interacting proteins 1 (RIPK1) and RIPK3 (30) and has been reported to act as a host defense mechanism when apoptotic death pathways are disabled by viral infection (31). For example, in a mouse model of influenza infection, necroptosis was shown to inhibit viral replication but with deleterious consequences to bronchial epithelial integrity (32). In humans, both H5N1 and H1N1 influenza–induced ARDS have been shown to be associated with necrotic cell death within the distal pulmonary epithelia (33, 34). Intriguingly, the accessory protein open reading frame 3a (Orf3a) expressed within the highly related SARS-CoV-1 genome has been recently demonstrated to induce necroptosis through activating RIPK3 (35). SARS-CoV-2 also expresses an Orf3a accessory protein (36). However, whether SARS-CoV-2 Orf3a promotes necroptosis has yet to be determined. Some MT-DNA release may also result from innate immune cell activation. For example, human neutrophils following activation extrude their MT-DNA due to an inability to complete mitophagy (37). Additionally, neutrophil extracellular traps (NETs), which have been found in the peripheral circulation of COVID-19 patients (38), are rich in MT-DNA (39). Neutrophils have also been identified as undergoing metabolic reprogramming in the context of SARS-CoV-2 (40). In addition to neutrophils, activated platelets also release mitochondria (41), which in turn can induce NET generation, possibly contributing to pulmonary prothrombotic complications observed in patients with severe COVID-19 illness (42). Additionally, MT-DNA has been shown to directly promote NETosis (43, 44). The source and mechanisms of MT-DNA release in response to SARS-CoV-2 infection are the subject of future investigation.

Multiple clinically established biomarkers, such as LDH, ferritin, CRP, and D-dimer, are currently being evaluated to assess the risk of clinical deterioration from a COVID-19 diagnosis (40, 45–49). However, as products of gene expression in response to both acute and chronic stimuli, they tend to be largely nonspecific measures of systemic inflammation, with the exception of LDH, a marker of cell death. Nevertheless, LDH can also be released by cells undergoing apoptosis, a predominantly antiinflammatory form of cell death (50). Ferritin is primarily produced by the liver and serves as an acute phase reactant. Similarly, D-dimer is a nonspecific test and provides us minimal insight into the underlying pathophysiology of the disease or events occurring at the cellular level. In contrast, there are accumulating observations that high amounts of MT-DNA release are specifically generated by necrotic cells (51). Additionally, high MT-DNA levels have been shown to be associated with acute lung injury, in multiple independent cohorts (12, 13, 52). In our cohort, although CYTB performed similarly to ferritin/D-dimer for mortality, it performed better than these markers in the context of identifying those at risk for an ICU admission or intubation, which are important resource utilization metrics. Moreover, we observed that MT-DNA levels were approximately 5-fold higher in COVID-19 patients who developed severe pulmonary dysfunction or eventually died, suggesting it is at least as sensitive a biomarker as other clinically established and exploratory indicators used in prediction models. To conduct plasma MT-DNA measurements, we employed a rapid PCR assay technique that takes about 60 minutes to complete because of the elimination of the DNA purification step. In resource-limited settings, this can be especially important given the current necessity to identify subjects at a higher risk of clinical deterioration. Additionally, PCR-based assays measuring MT-DNA tend to be less cost prohibitive and do not need specialized equipment, facilitating easy implementation. For the abovementioned reasons, we suggest that MT-DNA be investigated as an adjunct clinical biomarker in COVID-19. Nevertheless, whether MT-DNA levels are equivalent or better measures than routinely obtained laboratory measurements will require additional validation with independent cohorts.

Based on our data from this study, it is not possible to clearly determine if circulating MT-DNA contributes to the pathogenesis of COVID-19. Nevertheless, cell-free MT-DNA is itself a signatory marker for the release of other MT-DAMPs, which collectively drive proinflammatory cytokine expression through the engagement of pattern recognition receptors on innate immune cells (7). MT-DAMPs drive IL-6 expression by macrophages (29) and stimulate IL-8 release by neutrophils (9). Notably, IL-6 suppresses lymphopoiesis (53) while IL-8 promotes neutrophil release from the bone marrow (54) and, therefore, could possibly explain our observed correlation between MT-DNA levels and elevations in the neutrophil-to-lymphocyte ratio. We additionally noted a significant correlation between MAC and MT-DNA levels. Extracellular mitochondria have been reported to activate complement. Mannose-binding lectin has been observed to bind to cell-free mitochondria, resulting in C3 consumption in the peripheral blood of mice (55). Therefore, it is interesting to note that C3 consumption is a general sign of C3 convertase activity, which would be a prerequisite for the downstream generation of membrane attack components (56). Along those lines, complement activation has been implicated in the pathogenesis of COVID-19–related end organ damage, including acute lung injury (57, 58), with potential therapeutic implications (5, 59). Finally, MT-DAMPs, unlike other inflammatory markers linked to poor outcomes of COVID-19 patients, have been reported to directly cause acute pulmonary dysfunction and tissue damage (60). Administration of MT-DAMPs into the bloodstream or pulmonary airways of rodents promotes acute lung injury mediated by neutrophil chemotaxis and reactive oxygen species generation to mitochondrial formylated peptides (9, 14). In these studies, the formylated peptide receptor-1 inhibitor cyclosporine H was shown to inhibit MT-DAMP–mediated acute lung injury. Given ongoing clinical trials that target the effects of IL-6, complement activation and NETs in COVID-19 patients (3–5, 61, 62) future approaches that block necroptosis or MT-DAMP recognition could also be warranted.

Our study has several limitations. First, we were unable to concurrently enroll a COVID-19–negative group with severe respiratory disease. Our center, like many others, experienced a sharp decline in hospital presentations for illnesses other than COVID-19, making it difficult to collect samples for an adequately matched control group. Additionally, as our samples were drawn as a part of a prospective study of SARS-CoV-2 PCR-positive patients, any other adequately matched control group would not be entirely comparable, as not drawn concurrently. However, it is unlikely that MT-DNA levels are considerably different between patients with COVID-19 and acute respiratory diseases arising from other etiologies. For example, reports have shown that increased MT-DNA levels in critically ill patients are significantly associated with a higher risk of developing ARDS regardless of the underlying etiology (12, 13, 52). Nakahira et al. also showed that high level of plasma MT-DNA is a general marker of mortality risk in ICU patients (13). Additionally, more recent work from Huang et al. (52) shows that high plasma MT-DNA levels in ARDS patients are associated with increased risk for mortality regardless of admission diagnosis. Indeed, several emerging studies suggest that biomarkers may be no different in subjects with COVID-19–induced lung injury versus lung injury due to other etiologies (63). However, the strength of our study lies in an assay that can potentially identify subjects presenting with COVID-19 who are likely to develop adverse outcomes during their hospitalization, which becomes increasingly important when resources are constrained as has been unfortunately too common during this pandemic (64–66). Second, this study does not assess how MT-DNA correlates with viral load in COVID-19, which is especially important in the context of deciding when to intervene with targeted therapeutics (67–69). Therefore, further studies will be necessary to evaluate whether it may be a predictor of response to treatment (70). Third, decision-making in the ICU changed over the course of our enrollment. For example, there was a tendency toward intubating earlier in the course of the critical illness in the initial months of the COVID-19 pandemic. We also lack data to support objective measurement of disease severity at the time of presentation as can be reflected in SOFA, APACHE, or CURB65 scores. However, we identified MT-DNA levels as a predictor of severity utilizing mortality as a primary endpoint, then requirement of ICU admission and requirement for intubation as independent secondary endpoints. Additionally, we identified MT-DNA levels as a predictor of disease severity utilizing requirement of vasopressors and RRT while in the ICU as independent secondary endpoints of end organ dysfunction.

It also remains possible that our findings may not generally apply to all patients who contract COVID-19. Members of underrepresented minority communities are at significantly higher risk for COVID-19 infection as well as suffering its most severe outcomes. Our analysis was conducted in an urban medical center that serves a large African American community who may have a tendency to inherit MT-DNA variations that differ from other groups. This may be most exemplified in a recent report that shows certain point mutations within the MT-DNA of aging African Americans are associated with decreased pulmonary maximal inspiratory pressures (MIPs) (71). However, this study also found other point MT-DNA mutations in African Americans were associated with increased MIPs, suggesting a more complex picture for MT-DNA variation and its link to respiratory function. Nevertheless, this MT-DNA variation was not within the primer target sequences used in our assays. Finally, prior studies that analyzed other diseases that involve respiratory distress, such as ARDS and primary lung allograft dysfunction, on populations predominantly composed of Caucasians, and to lesser extent, underrepresented minority groups, demonstrated significant associations with cell-free circulating MT-DNA (12–14). Taken together with our findings, these reports support a robust association with MT-DNA levels and poor outcomes from COVID-19 infection that is likely applicable to all racial groups.

In summary, we demonstrate that MT-DNA measured early in the disease course can predict survival status, requirement for ICU-level care, the need for endotracheal intubation, as well as end organ dysfunction requiring vasopressors and RRT. We also show that MT-DNA levels are associated with exploratory biomarkers implicated in the pathogenesis of COVID-19–related morbidity and mortality. Further studies will be needed to discern the contribution of MT-DAMPs such as MT-DNA to COVID-19 pathogenesis, as well as to understand whether MT-DNA and other inflammatory mediators, such as activated complement, work synergistically to promote cellular injury.

## Methods

### Study design, settings, and participants.

This prospective cohort study utilized cell-free plasma samples that had been prospectively collected from 97 adult patients with confirmed COVID-19 presenting to the Barnes-Jewish Hospital from March 15, 2020, to April 24, 2020. Eligible participants included consented adults, greater than 18 years old, presenting with COVID-19 symptoms who received a COVID-19 test at the time of hospital presentation that resulted in a COVID-19 diagnosis. Diagnosis of COVID-19 was based on a positive nasopharyngeal swab test. Excluded participants were COVID-19–diagnosed patients who did not have plasma samples collected within 24 hours of time of hospital presentation.

### Outcome definition.

Subjects were followed through June 29, 2020. The primary outcome was mortality. The secondary outcomes included (a) the need for an ICU admission and (b) endotracheal intubation, as well as the development of end organ dysfunction while in the ICU requiring (c) vasopressors and (d) RRT. Subjects who were on dialysis prior to admission were excluded from the RRT outcome analysis. These outcomes were abstracted utilizing an honest broker system from electronic medical records.

### Sample collection and processing.

MT-DNA and other cytokines were measured in cell-free plasma of subjects within the first 24 hours of emergency department presentation. Blood samples were collected in EDTA-containing vacutainers (BD Biosciences) and subjected to 2 rounds of centrifugation to generate platelet poor plasma, first at 2500*g* for 20 minutes to generate plasma. The plasma was removed from vacutainers and centrifuged at 13,000*g* in sterile nuclease-free eppendorf tubes (Thermo Fisher Scientific) for 10 minutes to remove platelets. These platelet-poor specimens were then immediately stored at −80°C until further analysis. Concurrently measured clinical markers (i.e., CRP, ferritin, lactate dehydrogenase, D-dimer) were obtained from the electronic medical record.

### MT-DNA quantification.

MT-DNA quantification real-time PCR was performed in a Bio-Rad CFX-Connect machine using reaction mixture containing 0.1 μL of cell-free plasma, 10 μL iQ SYBR Green Supermix (Bio-Rad), 0.5 μL of 5 μM forward and reverse primers, and 8.9 μL H_2_O. Assays were performed in triplicate under the following conditions: 1 cycle at 95°C for 3 minutes, then up to 40 cycles at 95°C for 10 seconds and 55°C for 30 seconds, and then a melt curve was performed from 65°C to 95°C (0.5°C every 5 seconds). Primers for human cytochrome *b* (CYB; forward 5′-ATGACCCCAATACGCAAAAT-3′ and reverse 5′-CGAAGTTTCATCATGCGGAG-3′) and human cytochrome *c* oxidase subunit III (COX3: forward 5′-ATGACCCACCAATCACATGC-3′ and reverse 5′-ATCACATGGCTAGGCCGGAG-3′) were synthesized by Integrated DNA Technologies. Copy number was estimated by comparison to a real-time PCR standard amplification curve. To generate the standard curve, selected regions of human purified MT-DNA containing the target sequences for MT-CYTB (forward 5′-ATGACCCCAATACGCAAAAT-3′ and reverse 5′-GACGGATCGGAGAATTGTGT-3′) and MT-COX3 (forward 5′-ATGACCCACCAATCACATGC-3′ and reverse 5′-ATCAATAGATGGAGACATAC-3′) were amplified by PCR under the following conditions: initial denaturation at 94°C for 2 minutes, then up to 35 cycles of denaturation at 94°C for 30 seconds, annealing at 50°C for 30 seconds, and extension at 72°C for 1 minute. After amplification, MT-DNA was gel purified, extracted, and quantified by NanoDrop (Thermo Fisher Scientific). Serial dilutions were then used to calibrate the real-time PCR standard curves. Final results were expressed as copy number of MT-DNA, and their absolute values were converted to log_10_ before statistical analysis.

### Quantification of cytokines and complement activation.

Cell-free plasma was analyzed using a Cytokine 35-Plex Human Panel, which provides simultaneous measurement of 35 cytokines (Thermo Fisher Scientific). The assay was performed in accordance with manufacturer’s instructions with each subject sample performed in duplicate and then analyzed on a Luminex FLEXMAP 3D instrument. Internal validation control with every plate also was run in duplicate. Complement activation was assessed in cell-free plasma specimens (not previously thawed) using the soluble C5b-9 assay (BD Biosciences OptEIA Human C5b-9 ELISA set).

### Statistics.

Continuous variables were reported as median (interquartile range). The predictive value of each biomarker was expressed as AUC derived from the relative ROC curve. Spearman *r* coefficient was calculated to estimate the correlation between 2 continuous variables. Univariate logistic regression analysis was used to calculate the unadjusted ORs. Independent variables known to have a biological relationship with the outcomes were selected a priori based on existing literature for the multivariate logistic model to calculate the adjusted ORs (72–74). Model diagnostics were performed with Corrected Akaike Information Criterion, log likelihood, and model deviance. The MT-DNA was log (base 10) transformed prior to data analysis to better meet model assumptions. To facilitate interpretation for this variable, the OR estimates were raised to the 0.5 power, which is interpreted as the increase in the odds of the outcome for each 0.5 change in MT-DNA on the log scale. All the statistical analyses were calculated and all figures were prepared using GraphPad Prism 8.4.2 (GraphPad Software Inc.). For all the analyses, a *P* value less than 0.05 was considered significant.

### Study approval.

The study was approved by the Washington University School of Medicine Institutional Review Board (ID 202004091 and 202003085). Written informed consent was obtained from all subjects.

## Author contributions

HSK, AEG, and MI conceived the study; DS, MC, CG, HSK, and AEG performed methodology; DS, MC, and HSK ran software; JAO, AMR, and CG validated data; DS, MC, VP, MR, AMR, RA, LA, HSK, and AEG performed formal analysis; LM, DZ, SKS, and JHZ investigated; LM, DS, MC, HSK, and AEG curated data; DS, MC, HSK, and AEG wrote the original draft; DS, MC, LM, JH, CG, AMR, ZL, SKS, RH, MI, DK, HSK, and AEG reviewed and edited the draft; DS and MC performed visualization; HSK and AEG supervised, HSK and AEG administered the project, HSK, RRH, DK, and AEG acquired funding. Co–first authors DS and MC equally contributed to methodology, data, and software analysis. They also helped write and reviewed and edited the original draft. The decision to order them as listed was due to DS’s novel contribution to the PCR method used in this study.

## Supplementary Material

Supplemental data

Trial reporting checklists

ICMJE disclosure forms

## Figures and Tables

**Table 6:**
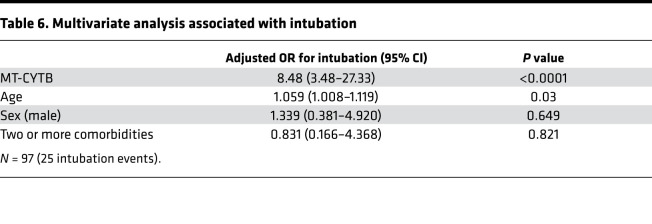
Multivariate analysis associated with intubation

**Table 1:**
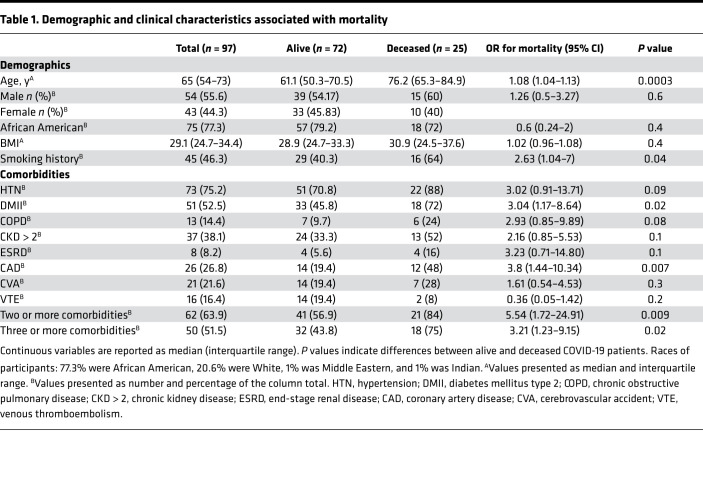
Demographic and clinical characteristics associated with mortality

**Table 2:**
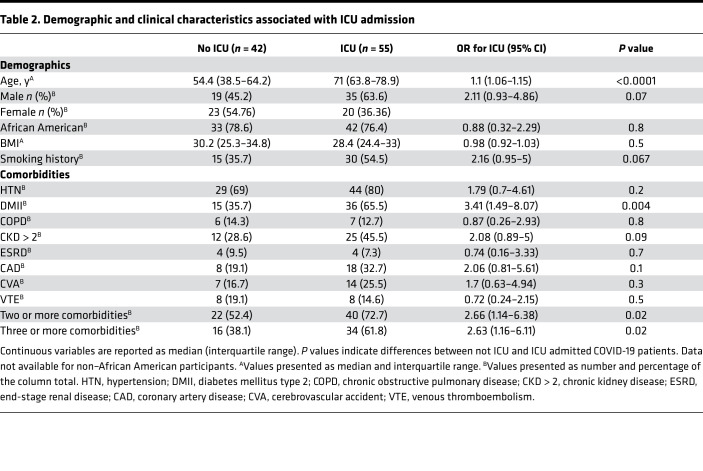
Demographic and clinical characteristics associated with ICU admission

**Table 3:**
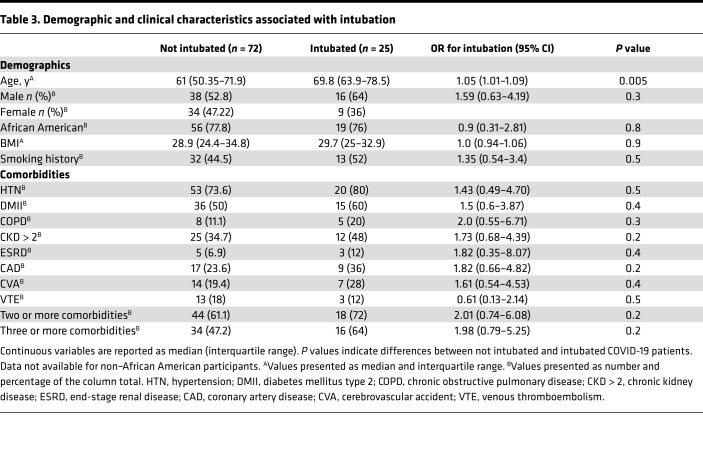
Demographic and clinical characteristics associated with intubation

**Table 4:**
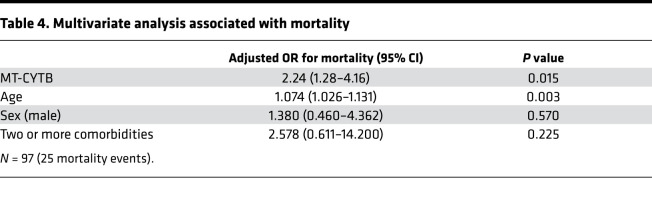
Multivariate analysis associated with mortality

**Table 5:**
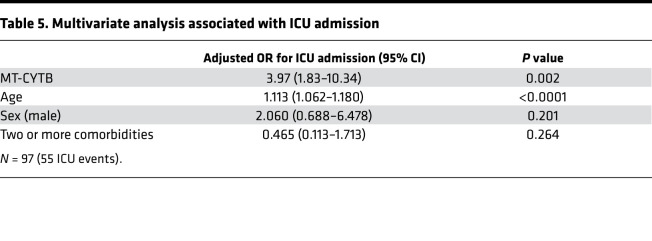
Multivariate analysis associated with ICU admission

## Version 1 01/14/2021

In-Press Preview

## Version 2 02/22/2021

Electronic publication
