# Supplementary material for: Circulating mitochondrial DNA is an early indicator of severe illness and mortality from COVID-19
Source: JCI Insight. 2021 Feb 22;6(4):e143299. doi: 10.1172/jci.insight.143299 (PMC7934921; doi:10.1172/jci.insight.143299)
Supplement: Supplemental data [file jciinsight-6-143299-s013.pdf]

Suppl. Fig. 1

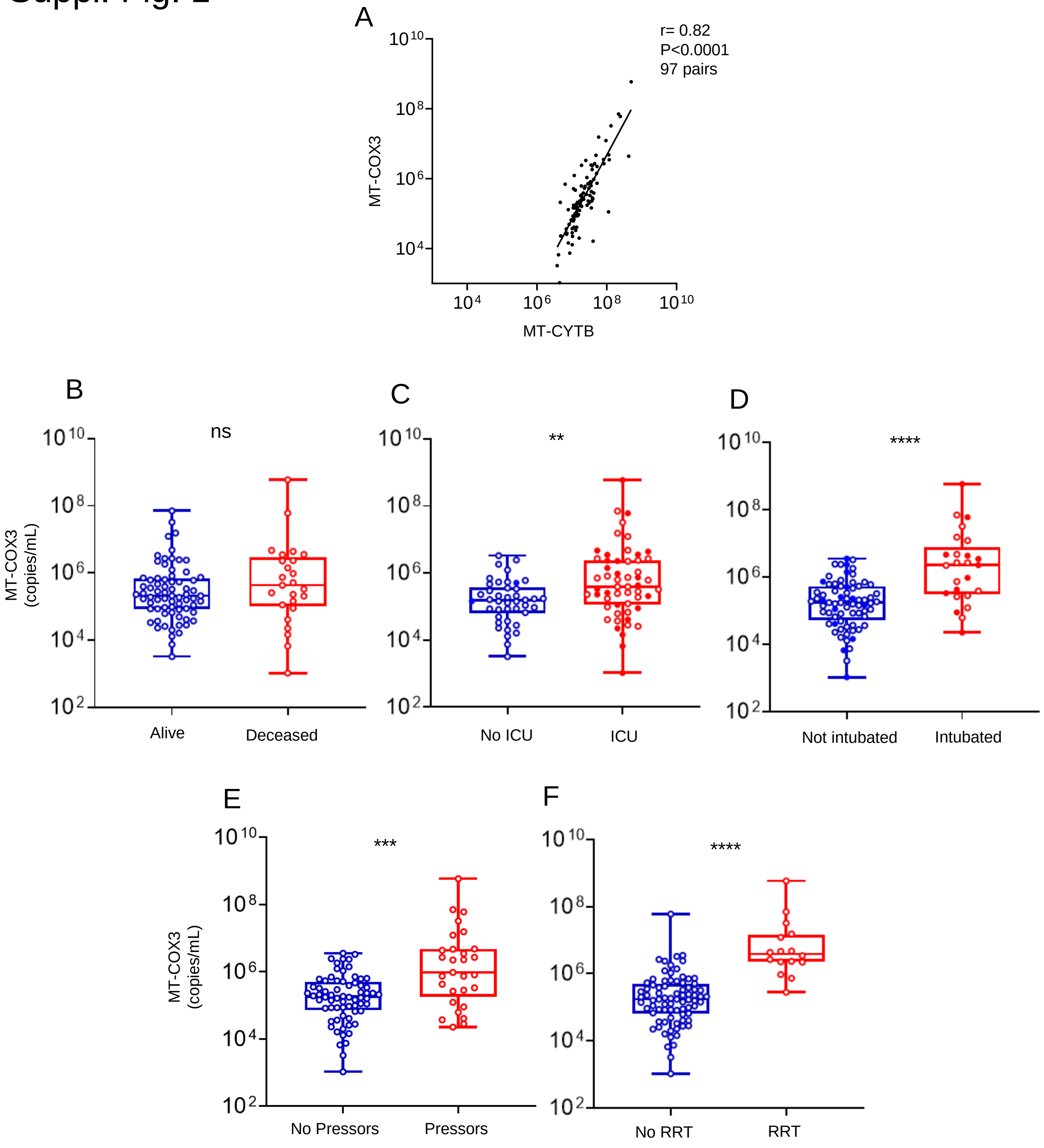

Suppl. Fig. 1. Comparable trends observed using MT-COX3 as an independent measure of MT-DNA. (A) Scatter plot showing the correlation between MT-CYTB and MT-COX3 measurements. The degree of correlation was assessed using Spearman`s Rank Correlation Coefficient test.

Box and whiskers plots of MT-COX3 levels in relation to (B) mortality status, (C) ICU admission, (D) intubation, (E) vasopressors and (F) Renal Replacement (RRT) in COVID-19 patients. In (C) and (D) the empty dots are for alive patients and the shded dots are for deceased patients. Statistical significance was determined using Mann-Whitney U Test (**P < .01,***P<.001 ****P < .0001).

Suppl. Fig. 2

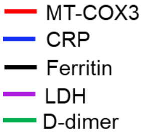

A

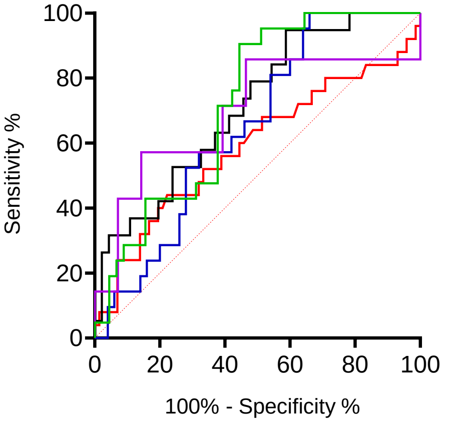

Mortality

	AUC 95% CI	P Value
**MT-COX3**	0.59 (0.44-0.73)	0.1
**CRP**	0.65 (0.52-0.77)	0.047
**Ferritin**	0.71 (0.58-0.84)	0.007
**LDH**	0.69 (0.43-0.95)	0.1
**D-Dimer**	0.72 (0.59-0.84)	0.004

B

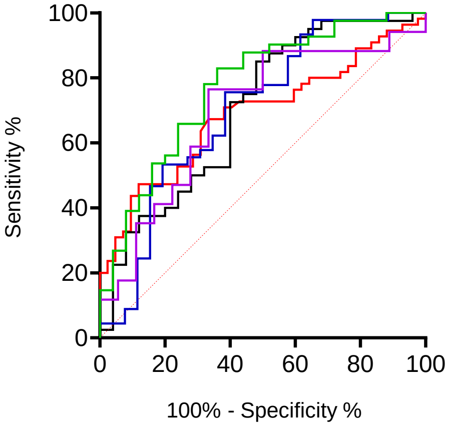

ICU

	AUC 95% CI	P Value
**MT-COX3**	0.69 (0.58-0.79)	0.0017
**CRP**	0.7 (0.56-0.83)	0.0052
**Ferritin**	0.7 (0.56-0.83)	0.0067
**LDH**	0.69 (0.51-0.87)	0.05
**D-Dimer**	0.77 (0.65-0.89)	0.0002

C

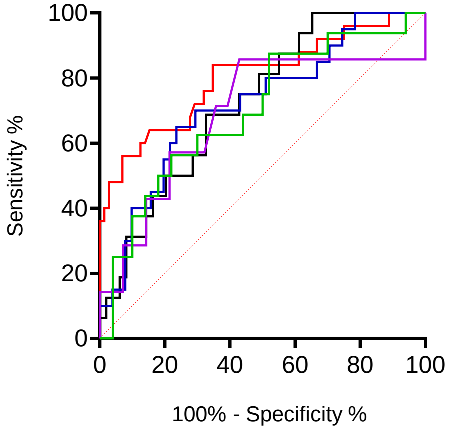

Intubation

	AUC 95% CI	P Value
**MT-COX3**	0.80 (0.69-0.91)	<0.0001
**CRP**	0.72 (0.58-0.85)	0.0042
**Ferritin**	0.72 (0.59-0.85)	0.007
**LDH**	0.69 (0.43-0.93)	0.1
**D-Dimer**	0.70 (0.54-0.84)	0.01

Suppl. Fig. 2. Comparison of the predictive values of MT-COX3 with current clinically used biomarkers of disease severity in COVID-19 patients. Blood samples for determination of biomarker levels were collected within 24 hours from hospital presentation. Receiver operating characteristic (ROC) curves in predicting the outcome (A) mortality, (B) admission to ICU and (C) Intubation based on MT-COX3 (red), reactive C protein (CRP) (blue), Ferritin (black), Lactic acid dehydrogenase (LDH) (purple) and D-Dimer (green) levels. Area under the curve (AUC) with 95% CI and P values for the different biomarkers are summarized in the corresponding tables.

Suppl. Fig. 3

A

Mortality

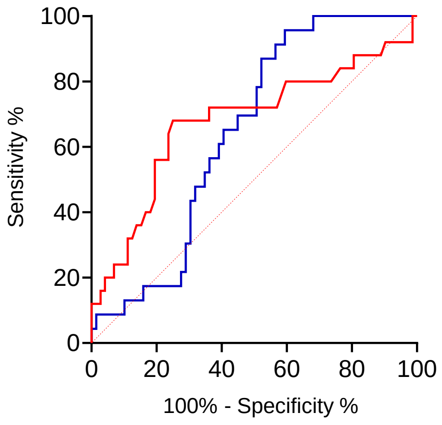

	AUC 95% CI	P Value
**MT-CYTB**	0.68 (0.54-0.81)	0.0088
**IL-6**	0.64 (0.53-0.75)	0.04

B

ICU

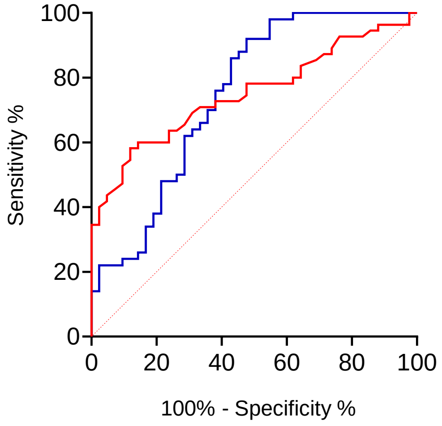

	AUC 95% CI	P Value
**MT-CYTB**	0.75 (0.65-0.85)	<0.0001
**IL-6**	0.75 (0.64-0.84)	<0.0001

C

Intubation

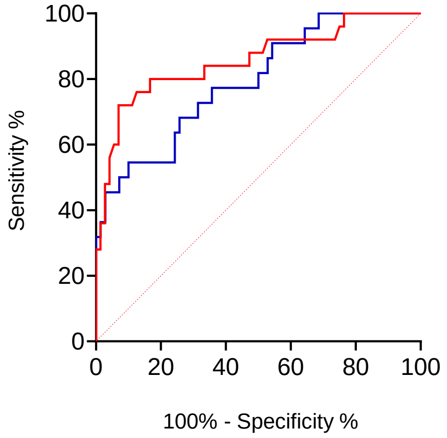

	AUC 95% CI	P Value
**MT-CYTB**	0.86 (0.76-0.95)	<0.0001
**IL-6**	0.79 (0.68-0.90)	<0.0001

Suppl. Fig. 3. Comparison between the predictive values of MT-CYTB and IL-6 over outcomes of disease severity in COVID-19 patients. Receiver operating characteristic (ROC) curves in predicting the outcome (A) mortality, (B) admission to ICU and (C) Intubation based on MT-CYTB (red) and IL-6 (blue). Area under the curve (AUC) with 95% CI and P values are summarized in the corresponding tables.
